# L-arginine Supplementation Protects Exercise Performance and Structural Integrity of Muscle Fibers after a Single Bout of Eccentric Exercise in Rats

**DOI:** 10.1371/journal.pone.0094448

**Published:** 2014-04-15

**Authors:** Yulia N. Lomonosova, Boris S. Shenkman, Grigorii R. Kalamkarov, Tatiana Y. Kostrominova, Tatyana L. Nemirovskaya

**Affiliations:** 1 Faculty of Basic Medicine, Lomonosov Moscow State University, Moscow, Russia; 2 Institute for Bio-Medical Problems, RAS, Moscow, Russia; 3 Emanuel Institute of Biochemical Physics, RAS, Moscow, Russia; 4 Department of Anatomy and Cell Biology, Indiana University School of Medicine-Northwest, Gary, Indiana, United States of America; National University of Singapore, Singapore

## Abstract

Eccentric exercise is known to disrupt sarcolemmal integrity and induce damage of skeletal muscle fibers. We hypothesized that L-arginine (L-Arg; nitric oxide synthase (NOS) substrate) supplementation prior to a single bout of eccentric exercise would diminish exercise-induced damage. In addition, we used N-nitro-L-arginine methyl ester hydrochloride (L-NAME; NOS inhibitor) to clarify the role of native NOS activity in the development of exercise-induced muscle damage. Rats were divided into four groups: non-treated control (C), downhill running with (RA) or without (R) L-Arg supplementation and downhill running with L-NAME supplementation (RN). Twenty four hours following eccentric exercise seven rats in each group were sacrificed and soleus muscles were dissected and frozen for further analysis. The remaining seven rats in each group were subjected to the exercise performance test. Our experiments showed that L-Arg supplementation prior to a single bout of eccentric exercise improved subsequent exercise performance capacity tests in RA rats when compared with R, RN and C rats by 37%, 27% and 13%, respectively. This outcome is mediated by L-Arg protection against post-exercise damage of sarcolemma (2.26- and 0.87-fold less than R and RN groups, respectively), reduced numbers of damaged muscle fibers indicated by the reduced loss of desmin content in the muscle (15% and 25% less than R and RN groups, respectively), and diminished µ-calpain mRNA up-regulation (42% and 30% less than R and RN groups, respectively). In conclusion, our study indicates that L-Arg supplementation prior to a single bout of eccentric exercise alleviates muscle fiber damage and preserves exercise performance capacity.

## Introduction

It is well documented that eccentric exercise can induce skeletal muscle damage through the disruption of sarcolemma and the break-down of cytoskeletal proteins (reviewed in [1], [2]). An increase in the number of damaged fibers was observed when dystrophin immunostaining, fluorescent dextran or Evans Blue dye were used to evaluate sarcolemmal integrity post-exercise [3], [4], [5]. Moreover, eccentric exercise can trigger degradation of the cytoskeletal and contractile proteins (reviewed in [1], [6], [7]) and prompt the appearance of desmin-negative muscle fibers [7], [8], [9]. Damage of the cytoskeletal and contractile proteins and impairments of the excitation-contraction system are believed to be the basis of declined post-exercise performance capacity [10], [11].

Despite the well-known effects of eccentric exercise on skeletal muscle damage, the mechanisms regulating these effects are not completely understood. Elevated calcium concentration observed in skeletal muscle hours and even days following eccentric exercise indicates its role in the regulation of activity of calcium-dependent cysteine proteases (calpains) and degradation of cytoskeletal and contractile proteins (reviewed in [1], [12], [13]). Recent data on the increased activity of calpains in response to very low calcium concentrations indicate that even a small increase in calcium concentration can have strong effects on protein degradation several days after an eccentric exercise bout [14], [15].

In addition to calcium, the activity of calpains can be regulated by other mechanisms including endogenous calpain inhibitor calpastatin [16], [17], and by nitric oxide (NO; [18], [19]). For example, Michetti and colleagues [20] reported that NO inhibited proteolytic activity of µ-calpain from the skeletal muscle *in vitro* through the S-nitrosylation of the active cysteine site. NO donor sodium nitroprusside counteracted effects of calcium ionophores on C2C12 cells preventing talin proteolysis, degradation of vinculin and protein loss [21]. A 10% stretching of C2C12 myotubes increased NO content and NOS activity, and these effects were potentiated by L-Arg and by calpain inhibitors and blocked by L-NAME [22].

Supplementation with nNOS substrate L-Arg can have a beneficial effect on skeletal muscle by alleviating muscle damage in mdx mice by inhibiting nuclear factor kappa B (NF-kB)/matrix metalloproteinase (MMP) cascades and increasing accumulation of utrophin [18], [23], [24]. L-Arg supplementation also can reduce skeletal muscle damage after ischemia-reperfusion [25] and reduce oxidative stress and inflammation after exhaustive exercise in young [26] and old [27] rats. The pharmacological effects of L-Arg supplementation are attributed to multiple factors, including increased NO production and bioactivity [28], decreased superoxide anions levels [29] and increased heme oxygenase expression [30].

Interestingly, some studies suggest that supplementation with NOS inhibitor L-NAME also can have a beneficial effect on skeletal muscle. For example, NO levels and calpain activity were significantly increased after repetitive eccentric contractions in rats and L-NAME supplementation decreased NO content, and diminished myofiber damage and leukocyte invasion three days post-exercise [31]. At the same time, combined treatment of mdx mice with deflazacort and L-Arg spared muscle from post-exercise injury induced regeneration while combined treatment with deflazacort and L-NAME failed to exert protective effects [23]. Intriguingly, the number of muscle fibers with damaged plasma membrane evaluated by Evans Blue dye incorporation was similar in both treatments and significantly smaller than in the placebo group [23]. This suggests that NO can exert its protective effects on the muscle via some additional mechanisms distinct from the reduced sarcolemmal permeability. One of the potential mechanisms could involve NO-dependent calpain inhibition and diminished degradation of the cytoskeletal and contractile proteins mediated by L-Arg supplementation.

We hypothesized that L-Arg supplementation prior to eccentric exercise would diminish exercise-induced damage of skeletal muscle fibers in rats. To our knowledge, our study is the first to investigate the effects of L-Arg supplementation on muscle damage after a single bout of eccentric exercise. We showed that L-Arg supplementation prior to eccentric exercise helps to prevent muscle fiber damage and preserve exercise performance capacity in rats. On the contrary, reduced NOS activity and NO content as a result of L-NAME supplementation prior to a single bout of eccentric exercise have not provided significant protection against muscle fiber damage in our study.

## Materials and Methods

### Animal Procedures

The experiments were carried out in accordance with the internationally accepted regulations and the rules of biomedical ethics and were approved by the Committee on Bioethics of the Russian Academy of Sciences (protocol 264, 25.02.2010). All animals were kept at 22°C in a light-controlled environment (12∶12 h light-dark cycle) with water and food available ad libitum. Fifty-six male Wistar rats (three months old, 254–276 g body weight range) were randomly assigned to one of the four groups with 14 animals/group: non-treated control (C), downhill running with (RA) or without (R) L-Arg supplementation and downhill running with L-NAME supplementation (RN). L-Arg (500 mg/kg of body weight) or L-NAME (90 mg/kg of body weight) were administered with drinking water continuously for forty eight hours prior to eccentric exercise. All rats were housed individually one per cage during L-Arg and L-NAME treatments. There was no spillage of water. The amount of water that the rats were drinking over 12-hour period was evaluated prior to the experiments. The concentrations of L-Arg and L-NAME in the drinking water were calculated based on the amount of water that rats were drinking over 24-hour period and on the body weight. An additional 12 rats were used for the determination of relative NO content in soleus muscle: 6 rats in the non-treated control (C) group and 6 rats in the L-Arg supplementation group (CA; 500 mg/kg of body weight). A detailed protocol used for the measurements of relative NO content is described below. A separate L-Arg supplementation group of rats was needed for the NO content determination assay since NO trap injection (diethyldithiocarbamate; DETC) that was required for the assay renders animals incapable of running. Twenty four hours following eccentric exercise, seven rats from each group were euthanized by overdose of sodium pentobarbital and soleus muscle was immediately dissected, weighed, divided into aliquots, frozen in isopentane cooled by liquid nitrogen, and stored at −85°C for subsequent analyses. The remaining animals (seven rats in each group) were used for the exercise performance capacity assessment.

### Eccentric Exercise

Eccentric exercise consisted of a single bout of downhill treadmill running at a speed of 20 m/min on a −16° incline for 40 min (Treadmill Simplex II, Columbus Instruments, USA). Rats were allowed to get acclimated to the treadmill one day before eccentric exercise testing by running at a speed of 5 m/min with an uphill grade of 2° for 15 min. Selecting a −16° incline for 40 min of eccentric exercise was based on the literature review and on our previous observations. Similar eccentric exercise conditions were used in a number of previously published studies [32], [33], [34], [35], [36], [37].

### Exercise Performance Capacity

Exercise performance capacity was tested on a treadmill twenty four hours after a single bout of eccentric exercise. Rats were run at a speed of 27 m/min with an uphill grade of 20° for 15 min. Running was maintained by electrical stimulation (40 V) of animals that slowed down or stopped. There were no significant signs of stress and in our opinion electrical shock did not compromise the validity of the exercise capacity assessments. Net running time was counted for 15 min of the assessment and the amount of work performed by each animal during the treadmill test was calculated based on treadmill speed and total running time for each rat (expressed in joule; J).

### Determination of Relative NO Content in Soleus Muscle

**Figure 1:**
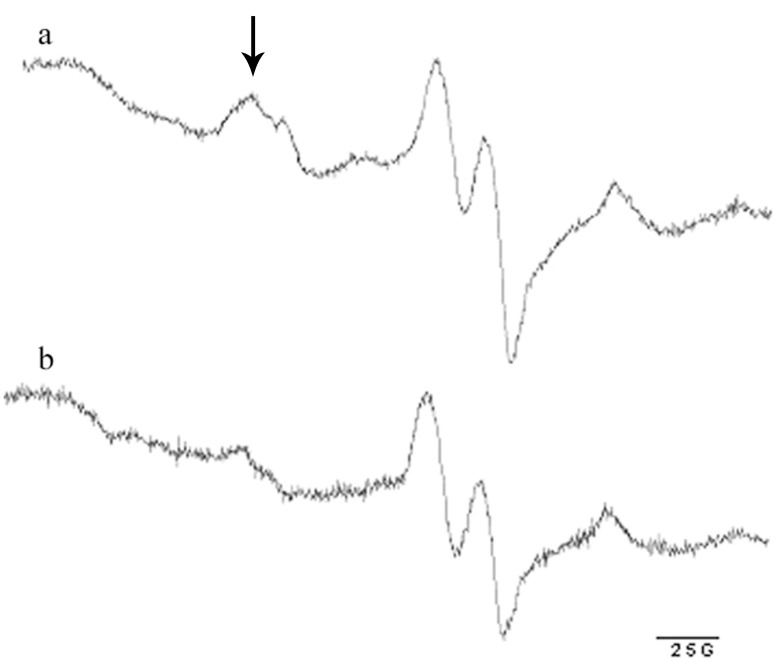
Representative picture of the typical EPR spectrum of sample from rat soleus muscle recorded after Fe-DETC administration. a, a representative spectrum from soleus muscle of rat from CA group after 48 hours of L-Arg administration with drinking water; b, a representative spectrum from soleus muscle of control rat without L-Arg administration. The spectrum component used to evaluate relative NO concentration is indicated by the arrow.

Relative NO content in soleus muscle after L-Arg administration was assessed in a separate group of rats by the standard spin trapping technique and electron paramagnetic resonance (EPR; [38], [39]). The L-Arg administered group (CA) received the drug according to the scheme for the RA group. Forty eight hours after L-Arg administration, control (C) and CA rats (6/group) received a diethyldithiocarbamate (DETC) injection (500 mg/kg), and then were intramuscularly injected with an aqueous solution of 29 mM FeSO4 and 116 mM sodium citrate (2 ml/kg). DETC was used for the spin trapping of NO since it forms iron nitrosyl complexes with a distinctive EPR spectrum when reacting with stationary NO present in the tissues. Thirty minutes later, the animals were anaesthetized with sodium pentobarbital, and soleus muscles were dissected and frozen in liquid nitrogen. EPR spectrum was recorded at the temperature of liquid nitrogen, frequency of 9.61 GHz, power of 2 mW, and modulation amplitude of 5 G on an EMX-8 EPR spectrometer (Bruker, Germany). The EPR signal is a superposition of the signal from the NO–Fe2+(DETC)2 complex and other paramagnetic centers detected in the tissues (Figure 1). The low-field spectrum component of the EPR signal was distinct and did not overlap with other signals. Therefore, relative NO content was estimated by the amplitude of the first component of the triplet-hyperfine structure of the EPR spectrum.

### Immunofluorescent Staining of Muscle Sections for Dystrophin

Transverse sections of soleus muscle were mounted on cork using OTC medium. Cryostat sections (10 µm thick) mounted to a glass slide were incubated for one hour with antibodies against dystrophin (Novocastra, NCL-DYSI; 1∶20), washed three times with buffer and then incubated for one hour with secondary goat anti-mouse antibodies (Molecular Probes; Alexa546, 1∶1000) at room temperature. The sections were examined and photographed with a Leica microscope at magnification x100. The proportion of the fibers with a damaged dystrophin layer relative to the total number of fibers was counted for each section.

### Protein Extraction and Western Blot Analysis

Skeletal muscle tissue (35 mg) was homogenized in ice-cold RIPA lysis buffer as previously described [40]. Samples were incubated for 20 min at 4°C and centrifuged for 10 min at 12,000 g. The protein content of the supernatants was quantified using a modified Lowry protocol (RC DC Protein Assay, Bio-Rad Laboratories, USA). Bovine albumin was used as a standard for concentration evaluation. The samples were diluted in Laemmli buffer. Total protein (40 µg/lane) was run on 12% SDS-PAGE and transferred to a nitrocellulose membrane (Bio-Rad Laboratories). Membranes were blocked overnight at 4°C with blocking buffer (4% nonfat milk powder, TBS pH 7.4, and 0.1% Tween-20) and incubated for 2 h at room temperature with the primary monoclonal antibodies against desmin (Santa Cruz; sc-14026, 1∶900) and primary monoclonal antibodies against GAPDH (Abcam; ab9484, 1∶900). In the preliminary experiments we verified that each antibody recognized just one protein band by performing Western blottings separately for each antibody. Since GAPDH (∼35 kDa) and desmin (∼55 kDa) bands were well separated in the gel it was possible in the subsequent experiments to evaluate them simultaneously on the same blot.

After three ten minute washes with TBS-Tween (TBS and 0.1% Tween-20), the membranes were incubated for one hour at room temperature with horseradish peroxidase-conjugated goat anti-mouse secondary antibodies (Bio-Rad Laboratories; 1∶200,000). The membranes were washed again in TBS-Tween three times for 10 min, incubated with Immun-Star HRP substrate (Bio-Rad Laboratories), and exposed to X-ray film (Kodak). Protein bands were quantified using densitometry scanning (GS-800, Quantity-One software, BioRad Laboratories). The relative content of desmin in each sample was determined by normalizing band intensities to the content of GAPDH in the same sample.

### RNA Analysis

Total RNA was extracted from 10 mg of frozen soleus muscle samples (7 rats per group) using an RNeasy Micro Kit (Qiagen, Germany). RNA samples were treated with proteinase K and DNase I. RNA concentration was determined at 260 nm. Isolated RNA in aqueous solution was frozen at −85°C for storage.

### Reverse Transcription

Reverse transcription was performed by incubating 1 µg of RNA, random hexamers d(N)6, dNTPs, RNase inhibitor, and MMLV reverse transcriptase for 60 min at 37°C.

### Quantitative PCR Analysis

For quantitative PCR analysis, 1 µl of cDNA was amplified in a 25 µl SYBR Green PCR reaction containing 1×Quantitect SYBR Green Master Mix (Syntol Moscow, Russia) and 10 µM of each forward and reverse primer. Sequences of the primers used in the current study are presented in Table 1. The annealing temperature was set according to the optimal annealing temperature of PCR primers. The amplification was monitored in real-time using the iQ5 Multicolor Real-Time PCR Detection System (Bio-Rad Laboratories). To confirm the amplification specificity, the PCR products from each primer pair were subjected to a melting curve analysis. Relative quantification was performed based on the threshold cycle (CT value) for each of the PCR samples [41]. Initially several housekeeping genes were evaluated for the normalization: GAPDH, cyclophilin A, β-actin, Rn18s, Rn28s and RPL19. Normalization to the level of expression of GAPDH, cyclophilin A and β-actin showed similar results (data not shown). β-actin was chosen for the normalization of all quantitative PCR analysis experiments in the current study.

**Table 1:** Primers used for QRT-PCR study.

Gene Description	Forward Primer	Reverse Primer	Accession No. GeneBank
**β-actin**	5′-TCATGAAGTGTGACGTTGACATCC-3′	5′-GTAAAACGCAGCTCAGTAACAGTC-3′	NM_031144.2
**µ-calpain**	5′-CATGGCTAAGAGCAGGAAGG-3′	5′-CGAAGTCTGCAGGTCTAGGG-3′	NM_019152.2
**GAPDH**	5′-ACGGCAAGTTCAACGGCACAGTCAA-3′	5′-GCTTTCCAGAGGGGCCATCCACA-3′	NM_017008.2
**HSP70**	5′-CCTCCGATTTCAGCTCAG-3′	5′-CGAAGGCGTAGAGATTCCAG-3′	NM_153629.1
**HSP90**	5′-GAGGCAGAGGAAGAGAAAGG-3′	5′-ATGGGCTTCGTCTTATTCAG-3′	NM_001004082.3
**MAFbx**	5′-CTACGATGTTGCAGCCAAGA-3′	5′-GGCAGTCGAGAAGTCCAGTC-3′	NM_133521.1
**MuRF-1**	5′-GCCAATTTGGTGCTTTTTGT-3′	5′-AAATTCAGTCCTCTCCCCGT-3′	NM_080903.1
**nNOS**	5′-AGTCCCCTGCTTCGTGAGAG-3′	5′-CACCCGAAGACCAGAACCAT-3′	NM_052799.1
**ubiquitin C**	5′-CACCAAGAAGGTCAAACAGGA-3′	5′-GCAAGAACTTTATTCAAAGTGCAA-3′	NM_017314.1

### Statistical Analysis

Statistical Analysis was performed using the REST 2009 v.2.0.12 and Origin Pro v.8.0 SR5 programs at the significance level set at 0.05. Comparisons of group pairs (C and CA) were made using nonparametric Mann-Whitney rank-sum test. The significance of the differences between four treatment groups (C, R, RA, RN ) was evaluated using the nonparametric version of the Newman-Keuls test for multiple comparisons. Results are given as median and interquartile range (0.25–0.75).

## Results

### Effect of L-Arg Supplementation on Tissue Content of NO

Representative pictures of typical EPR spectra from sample of rat soleus muscle recorded after Fe-DETC administration are presented in Figure 1. After L-Arg treatment NO content in muscle of the CA group increased by 46% (p<0.05) when compared with the control (1.46 (1.20–1.74) and 1.0 (0.9–1.13) arbitrary units, respectively).

### Effects of L-Arg and L-NAME Supplementation on the Dystrophin Layer Integrity and Content of Desmin

**Figure 2:**
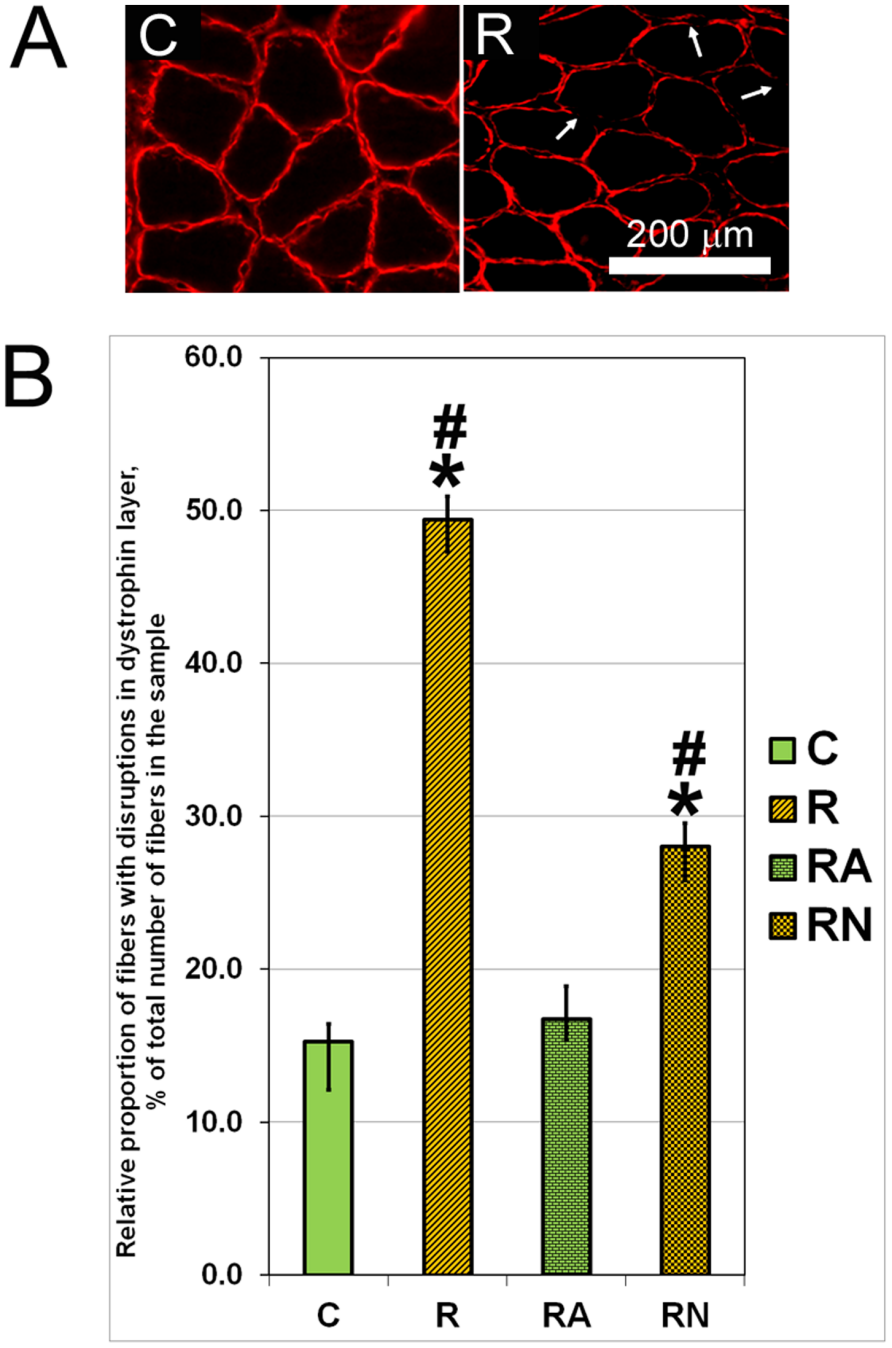
Representative picture of the immunostaining of samples from soleus muscles of C and R rats with antibodies against dystrophin (A) and proportion of muscle fibers with disruptions of immunostaining in the dystrophin layer (B) after normalization for the total number of fibers in each sample. Arrows in A indicate disruptions of immunostaining in the dystrophin layer. N = 7. *indicates a significant difference from control, p<0.05; #indicates a significant difference from RA, p<0.05.

Representative pictures of muscle fibers from soleus muscle of control rats without disruptions of dystrophin layer as well as fibers from soleus muscle of R rats with the disruptions of immunostaining in the dystrophin layer are presented in Figure 2A. The proportion of the fibers with disruptions in the dystrophin layer in the R group was 3.26- and 1.87-fold higher (p<0.001) than in the RN and control groups, respectively (Figure 2B). The proportion of the fibers with disruptions in the dystrophin layer in the RA group was similar to those found in the control group (Figure 2B). Therefore, L-Arg treatment effectively counteracted dystrophin layer degradation after a single bout of eccentric exercise (Figure 2).

**Figure 3:**
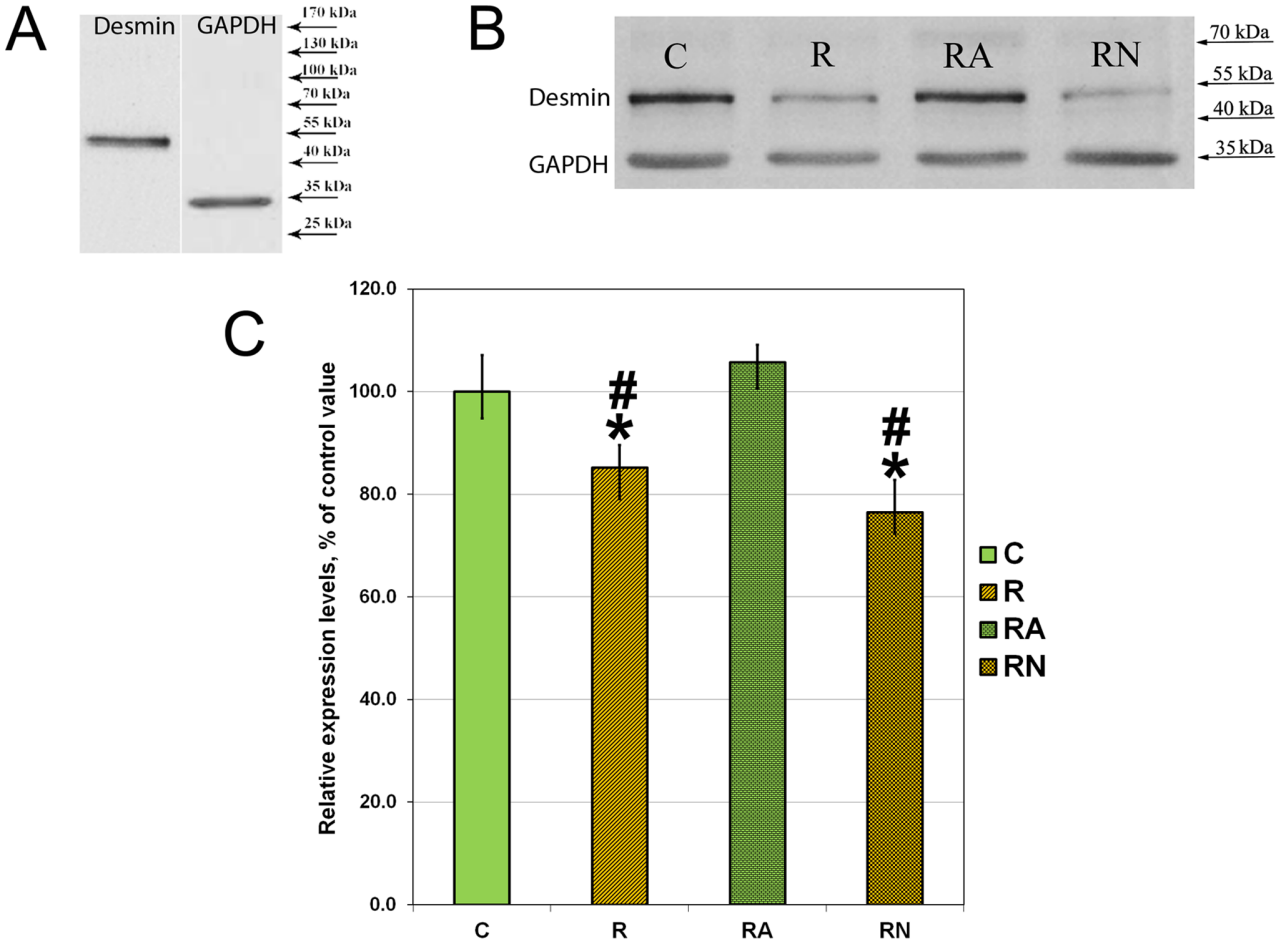
Representative picture of the preliminary evaluations of antibodies (A) and Western blotting of samples from soleus muscles of C, R, RA and RN rats with antibodies against desmin and GAPDH (B), and relative desmin protein expression level (C) after normalization for the GAPDH protein expression level in each sample. N = 7. *indicates a significant difference from control, p<0.05; #indicates a significant difference from RA, p<0.05. This Figure was previously published as proceedings in [67].

Desmin content was evaluated in samples of soleus muscles from C, R, RA and RN rats using Western blotting. In the preliminary experiments we performed Western blots separately with GAPDH and desmin antibodies and verified that each antibody recognized a single protein band (Figure 3A). Since GAPDH (∼35 kDa) and desmin (∼55 kDa) bands were well separated in the gel in the subsequent experiments we evaluated them simultaneously on the same blot. A representative picture of Western blotting of soleus muscles from C, R, RA and RN rats is shown in Figure 3B. Desmin content was significantly lower in the R and RN groups when compared with the control group (by 15% and 25%, respectively; Figure 3B; p<0.05). L-Arg treatment prevented desmin content decrease after a single bout of eccentric exercise (Figure 3C).

### Effects of L-Arg and L-NAME Supplementation on Exercise Performance Capacity

**Figure 4:**
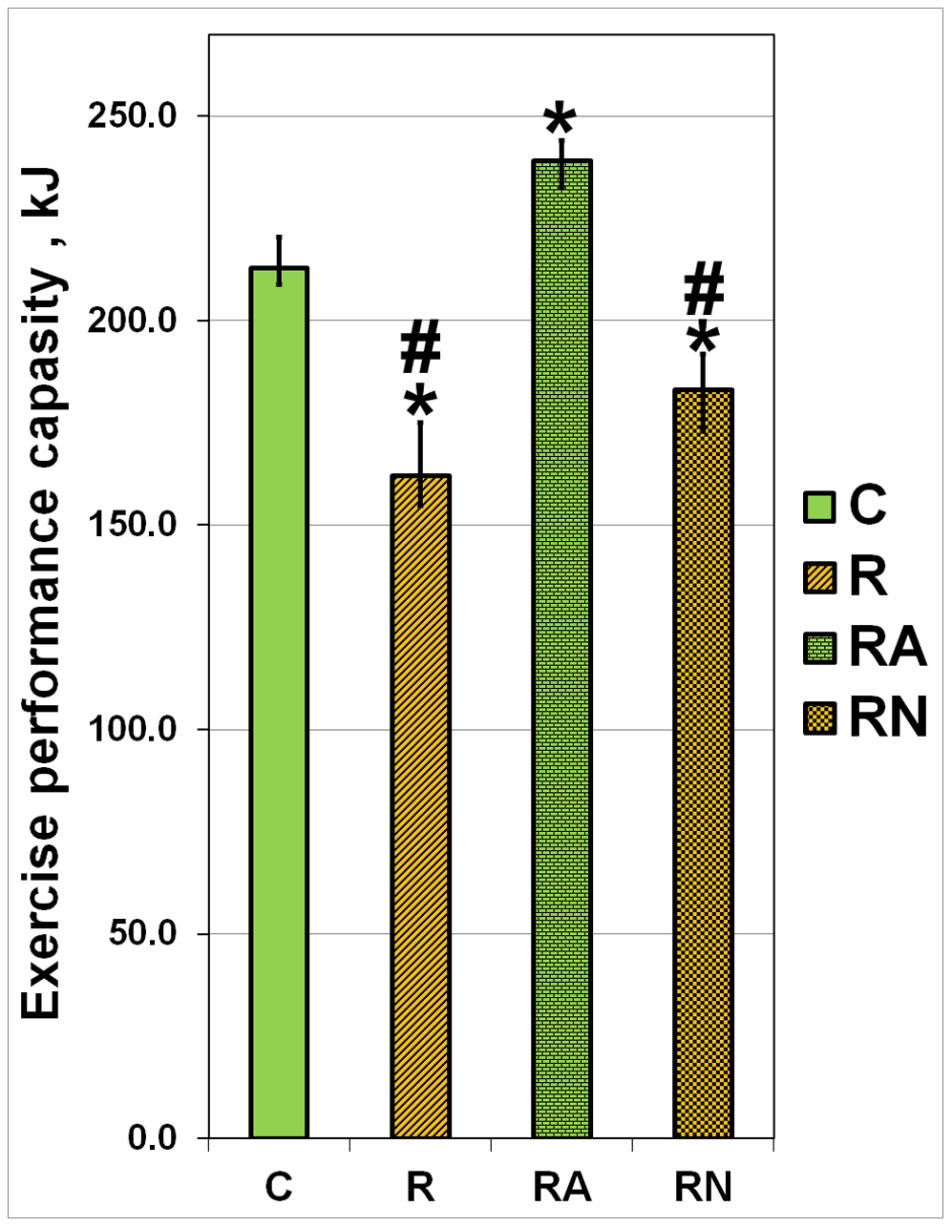
Exercise performance capacity of C, R, RA and RN rats twenty four hours post a single bout of eccentric exercise. N = 7. *indicates a significant difference from control, p<0.05; #indicates a significant difference from RA, p<0.05. This Figure was previously published as proceedings in [67].

The exercise performance capacity was evaluated in C, R, RA and RN rats twenty four hours after a single bout of eccentric exercise (Figure 4). The RA rats showed 12% and 47% higher exercise performance capacity when compared with C and R animals, respectively (p<0.05). The exercise performance capacity of RN animals was similar to the performance capacity of R animals and significantly lower than the capacity of the control animals (Figure 4).

### Effects of L-Arg and L-NAME Supplementation on the Level of mRNA Expression of Genes Involved in NO Generation, Protein Degradation and Cytoprotection

**Figure 5:**
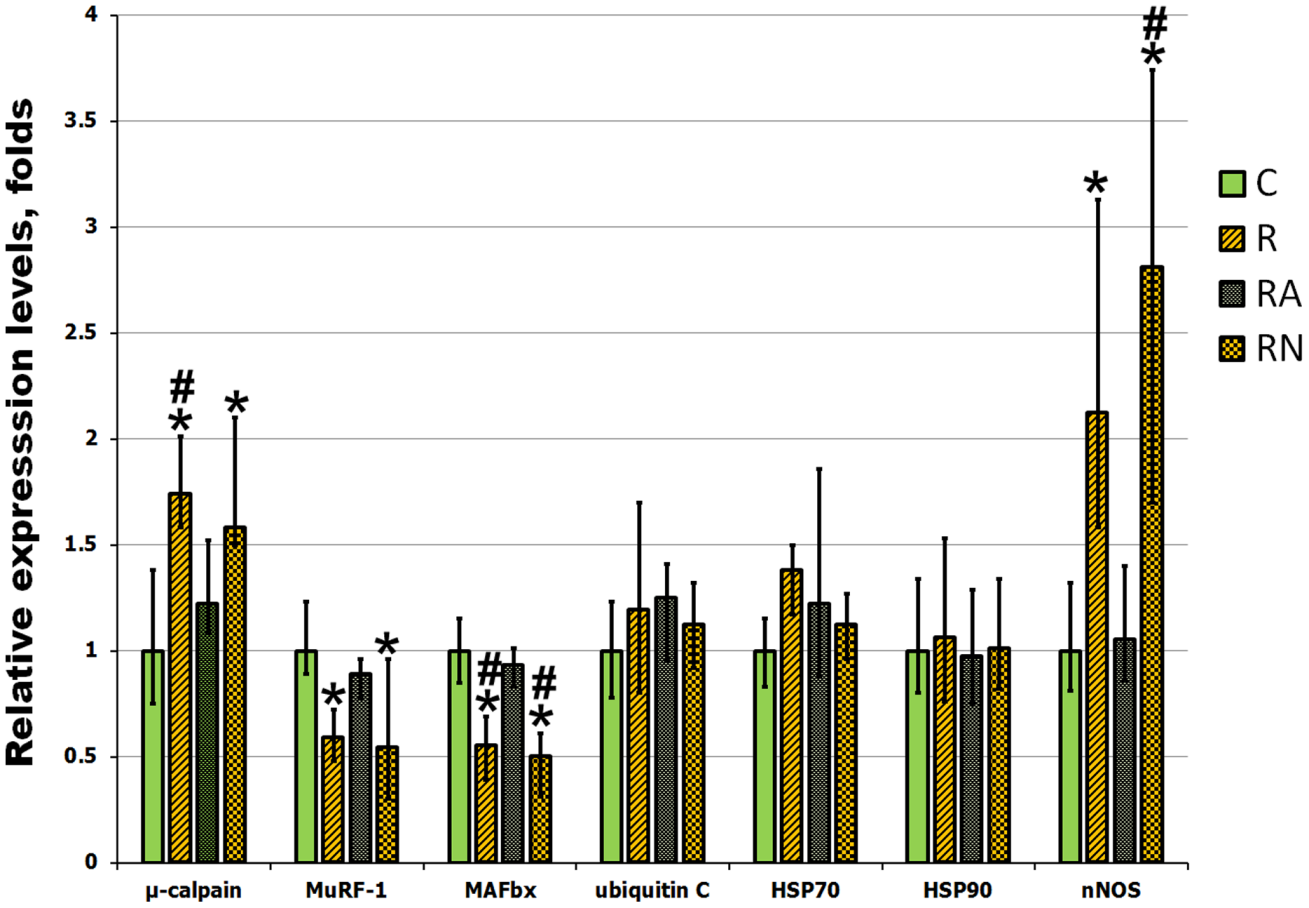
Evaluation of gene expression in soleus muscles of C, R, RA and RN rats. mRNA expression of genes involved in NO generation, protein degradation and cytoprotection was evaluated by quantitative PCR analysis. N = 7. Values are normalized to the expression of β-actin in each sample. *indicates a significant difference from control, p<0.05; #indicates a significant difference from RA, p<0.05.

The evaluation of the levels of mRNA expression of genes involved in protein degradation showed that a single bout of eccentric exercise (R group) resulted in a significant increase in the expression of µ-calpain (Figure 5; p<0.05). At the same time there was no change in the expression of ubiquitin C and surprisingly there was a decrease in the expression of E3 ligases muscle-specific RING finger protein 1 (MuRF-1) and muscle atrophy F-box (MAFbx) (Figure 5; p<0.05). mRNA expression of the cytoprotective heat shock proteins (HSP) HSP70 and HSP90 has not shown statistically significant differences after a single bout of eccentric exercise when compared with control non-exercised rats (Figure 5). mRNA expression of nNOS was significantly increased after a single bout of eccentric exercise (R group) when compared with the control (Figure 5; p<0.05).

L-NAME supplementation prior to a single bout of eccentric exercise (RN group) had not changed mRNA expression of the analyzed genes (Figure 5). In contrast, L-Arg supplementation prior to a single bout of eccentric exercise (RA group) had a substantial effect on gene expression, bringing the levels of post-exercise mRNA expression similar to those found in the non-exercised control rats (Figure 5).

## Discussion

Eccentric exercise is recognized for its ability to induce skeletal muscle damage through the disruption of sarcolemma and the break-down of cytoskeletal proteins [6], [42]. However, the factors regulating these effects are not completely understood. The current study was designed to elucidate the molecular mechanisms regulating the damaging effects of eccentric exercise on skeletal muscle fibers. Our findings showed that L-Arg supplementation prior to a single bout of eccentric exercise alleviates muscle fiber damage and maintains exercise performance capacity by decreasing the proportion of fibers with a damaged dystrophin layer and by preserving desmin content.

We used soleus muscle for our study since it was often used in the past for examining the effects of eccentric exercise-induced muscle damage [43], [44]. In rats soleus muscle consists of almost all slow fibers. Slow fibers have larger number of mitochondria, better developed antioxidant machinery and are more resistant to the effects of exercise-induced oxidative stress when compared with fast fibers. In comparison with fast fibers, slow fibers have higher pre- and post- exercise mRNA expression levels of TNF-α, calpains 1 and 2, MuRF-1, atrogin-1, caspase-3, Bcl-2, and Bax [45]. In vitro fiber-type sensitivity to mechanical strain is type IIa/IIx>type IIa = type I and therefore fiber type composition might determine muscle’s susceptibility to eccentric damage [46]. We were interested in the effects of L-Arg and L-NAME on mRNA expression of calpains, MuRF-1 and atrogin-1. We used rat soleus muscle in our study since in the past soleus muscle was shown to respond to exercise by highly up-regulated mRNA expression of these genes [45].

Eccentric exercise is known to decrease exercise performance, which is largely attributed to the delayed onset of muscle soreness resulting from damage of the cytoskeletal proteins [6] and to the reduction in the excitation-induced calcium release from the sarcoplasmic reticulum [11], [47]. Generally, protein degradation peaks at twenty four to forty eight hours post-exercise and the recovery of post-eccentric exercise performance capacity takes more than twenty four hours [47], [48], [49]. Interestingly, a short-term sprint exercise or endurance cycling in trained subjects does not trigger the detrimental damage of cytoskeletal proteins described above for the eccentric exercise [13], and muscle performance is quickly restored within one hour post-exercise.

Evaluations of the disruptions in dystrophin layer [3] and degradation of desmin [7], [11], [50], [51]were previously used for the analysis of muscle damage caused by exercise thus we used these methods for the damage assessment in our study. The changes in desmin following eccentric contractions were first documented by Lieber and colleagues [52] by showing loss of desmin immunostaining in 20% of fibers one day after a bout of eccentric exercise. The changes in desmin immunostaining were still observed three days post exercise. Zhang and colleagues [7] showed that the changes in desmin could be detected as early as 30 min after eccentric contractions. In agreement with the previously published results, our experiments demonstrated that exercise performance capacity was decreased in rats twenty four hours after eccentric exercise and this decrease was accompanied by the degradation of cytoskeletal proteins.

L-Arg treatment preserved exercise performance capacity and prevented dystrophin layer degradation and decrease of desmin content after a single bout of eccentric exercise. Our experiments showed that exercise performance capacity measured twenty four hours after a single bout of eccentric exercise was higher in L-Arg supplementation rats when compared with control non-treated non-exercised rats. We attributed this finding to the preservation of dystrophin and desmin content after eccentric exercise as well as to adaptive changes in the structure of the sarcomeres related to the previously described post-exercise conditioning of muscle [53], [54] and improved mitochondrial calcium homeostasis [55].

L-Arg is a NOS substrate and L-Arg treatment in our experiments caused a 46% increase in muscle NO content, suggesting that L-Arg-mediated preservation of exercise performance capacity after eccentric exercise is in part mediated by elevation of NO content. Previously published data demonstrated that both nNOS and eNOS activities were up-regulated in gastrocnemius muscles of rats after a 45-min bout of exhaustive treadmill running and NOS activity was decreased after L-NAME treatment [56]. Increased NOS activity and NO content were also reported in response to electrical stimulation-induced contractions of isolated muscle fibers in vitro [57].

In our experiments exercise induced an up-regulation of nNOS mRNA expression level in soleus muscles when compared with muscles of control non-exercised rats. Blocking of NOS activity by L-NAME treatment had no significant effect on the post-exercise levels of nNOS mRNA expression when compared with muscles of control non-exercised rats. This suggests that exercise-induced up-regulation of NOS activity and increased NO content are not the primary regulatory stimuli for the up-regulation of nNOS mRNA expression. To the contrary, L-Arg treatment averted exercise-induced up-regulation of nNOS mRNA expression. From these data we concluded that abundant availability of NO substrate (L-Arg) in combination with increased exercise-induced NOS activity blocked signaling stimuli required for the exercise-induced up-regulation of nNOS mRNA expression in RA rats. One of these stimuli might be exercise induced nuclear NF-κB signaling mediated by activated calpain-3 [58].

The role of calpains in the degradation of cytoskeletal proteins during eccentric exercise and the effect of calcium concentrations on this process has been previously described (reviewed in [7], [49]). Skeletal muscles express µ-calpain and m-calpain as well as muscle-specific calpain 3. At resting conditions, calpains are predominantly present in the full-length, non-autolysed/inactivated forms and the activation of calpains requires autolysis that is tightly regulated by calcium concentrations (reviewed in [49]). Different modes of exercise can have different effects on the regulation of amplitude and duration of calpain activation. For example, µ-calpain and calpain 3 are not activated by sprint exercise bouts or by endurance cycling in trained subjects [13]. In contrast, calpain-3, but not µ-calpain, was activated twenty four hours after a single bout of eccentric exercise but showed no changes in the activity immediately and three hours post-exercise [59]. At the same time, Zhang and colleagues [7] reported that µ-calpain but not calpain-3 was activated thirty minutes after eccentric contractions of mouse EDL muscle *in vitro*. Increased mRNA expression of m-calpain was reported in healthy males six and twenty four hours after a single bout of eccentric exercise [60].

Interestingly, calpains activation is responsive to very small increases in cytoplasmic calcium concentrations. For example, calpain-3 becomes activated even when exposed for a prolonged period of time to resting cytoplasmic calcium concentrations that are only two- to four-fold higher than normal (reviewed in [49]) supporting the role for calpain-3 in post-eccentric exercise remodeling of sarcomeres. Moreover, exercise-induced µ-calpain activation could have an effect on exercise-induced decrease in excitation-contraction coupling by regulated proteolysis of junctophilins [11]. We hypothesized that elevated NO content in soleus muscle of RA rats after L-arg supplementation can have an effect on both the expression and activity of calpains in post-exercised muscle. The ability of L-arg supplementation to block exercise-induced up-regulation of µ-calpain mRNA expression was confirmed in the current study. Our data on the decrease in exercise-induced degradation of cytoskeletal proteins suggests that L-arg supplementation might also interfere with exercise-induced up-regulation of calpain activity. In the future studies we plan to address this question directly by assessing whether L-arg supplementation blocks exercise-induced up-regulation of calpain activity.

When testing calcium-induced activation of calpains it is very important to use physiologically relevant calcium concentrations to perform the experiments. For example, eccentric contractions of skeletal muscle in mice increased calcium concentrations from 100 nM in resting muscle to 250 nM post-contraction [12]. Under these conditions NO regulates native calcium-dependent activity of calpains (reviewed in [49]). Some of the published studies used very high non-physiological calcium concentrations to evaluate the effects of NO on calcium-dependent activity of calpains and therefore the conclusions based on the results of these studies might be inaccurate. For example, Sakurai and colleagues [31] recently used very high millimolar concentrations of calcium to show that treatment with NOS inhibitor L-NAME decreased calpain activity after repetitive eccentric contractions in rats and diminished myofiber damage three days post-exercise. These data do not correlate with the results of the current study and the data published by Archer and colleagues [23]. Our studies showed that L-NAME supplementation prior to the eccentric contractions decreased the number of fibers with disruptions in the dystrophin layer but had no effect on the exercise-induced decrease of desmin content or changes in the gene expression. The differences in the experimental approach could have affected the disagreements in the results between studies from different laboratories. Further evaluation of the L-NAME effect on exercise-induced muscle damage is required to clarify disagreements in the data described above.

In addition to the calpains we evaluated regulation of the expression of ubiquitin-proteasome pathway proteins in response to the eccentric exercise. mRNA expression of MAFbx and MuRF-1 was substantially lower in the R and RN groups relative to the unexercised control. L-arg supplementation blocked exercise-induced down-regulation of MAFbx and MuRF-1 mRNA expression. These results are in agreement with previously published data from other laboratories [61], [62], [63]. For example, Nedergaard and colleagues [62] reported down-regulation of MAFbx in response to eccentric exercise. mRNA expression of MAFbx was also down-regulated at three, six and twenty four hours post lengthening contractions of quadriceps muscle in humans [61]. In our experiments mRNA expression of ubiquitin C was similar in all tested groups of rats. The lack of activation of the ubiquitin-proteasome pathway after eccentric exercise suggests that activation of the calpains might be the primary cause of the eccentric exercise-induced degradation of cytoskeletal proteins.

Induction of HSPs is an important adaptation of skeletal muscle to stressful conditions including exercise-induced stress (reviewed in [64]). Surprisingly, our experiments detected no changes in mRNA expression of HSP70 and HSP90 in any of the tested groups of rats. There was a trend for the increased mRNA expression of HSP70 but it has not reached statistically significant levels. These data differ from the previously published data that reported a 1.5-fold increase of HSP70 mRNA expression in soleus muscle of rats in response to downhill running when compared with horizontal plane running [65]. Mikkelsen and colleagues [66] also reported increased mRNA expression of HSP70 after a single bout of eccentric contractions in humans. Therefore, further evaluation of the effects of eccentric exercise on HSPs expression is required to clarify disagreements in the data described above. The differences in the experimental approach could have influenced the disagreement in the data.

In conclusion, the major findings of the current study are that L-Arg supplementation prior to a single bout of eccentric exercise alleviates muscle fiber damage and preserves exercise performance capacity. These effects are mediated by the increase of muscle NO content and are validated by decreased expression of calpain, reduced number of damaged muscle fibers and reduced loss of desmin content in muscle fibers in L-Arg supplemented group when compared with exercised rats without supplementation.
